# Characterization of a Surface Hydrogen Charging Product Affecting the Mechanical Properties in 2205 Duplex Stainless Steel

**DOI:** 10.3390/ma12101682

**Published:** 2019-05-23

**Authors:** Bo Kan, Zixuan Yang, Jinxu Li

**Affiliations:** Corrosion and Protection Center, Institute for Advanced Materials and Technology, University of Science and Technology Beijing, Beijing 100083, China; kanbo10008@163.com (B.K.); yangzixuan@nub.edu.cn (Z.Y.)

**Keywords:** characterization, thermal analysis, X-ray diffraction, secondary ion mass spectrometry, mechanical properties, hydride

## Abstract

When 2205 duplex stainless steel (DSS) is immersed in simulated seawater under high hydrostatic pressure, or in an electrochemically hydrogen charged state, a spindle-shaped product is found in the ferrite phase that seriously deteriorates the mechanical properties of 2205 DSS. This paper systematically studied the composition, structure, and properties of the hydrogen charging product. The results of a slow strain rate tensile test show that the hydrogen charging product evidently reduces the elongation of 2205 DSS, and microcracks mainly initiate at the interface between the hydrogen charging product and the ferrite matrix at either a low or a high strain rate. However, the elongation recovers to that of the hydrogen free sample after heating the sample at 300 °C for 0.5 h. The nano-hardness and reduced modules of the product are higher than those of the ferrite and austenite phases. An element analysis by energy dispersive spectroscopy (EDS) and secondary ion mass spectrometry (SIMS) indicates that the Ni and H contents in the hydrogen charging product are higher than in the normal ferrite area, and X-ray diffraction shows the characteristic peak of iron hydride at 40.07°. Moreover, a differential scanning calorimeter (DSC) test demonstrated that the phase decomposition temperature of the product is 268 °C, which coincides with the fact that it dissolves at a high temperature caused by the focused electron beam during transmission electron microscopy (TEM) analysis. All experimental results indicate that the hydrogen charging product is a hydride of FeH or (Fe, Ni)H.

## 1. Introduction

With the increasing demand for energy, the focus on oil and gas resource exploitation has shifted from the land to the deep ocean in recent years, and the exploitation depth has gradually increased. Submarine pipelines are exposed to harsh environmental conditions [1,2,3,4], such as high hydrostatic pressure, and cathodic protection is often applied during the service process. However, cathodic protection elevates the hydrogen content in the material [5,6,7,8,9], while high hydrostatic pressure will also cause a rise in the hydrogen content [10,11]. Does the dual effect lead to any changes in the microstructure and mechanical properties of the material? Although the surface of the pipeline steel is always coated by a protective layer in service [12,13,14,15,16,17], it is still necessary to understand how the material itself changes at high hydrogen concentration. Recently, we found that a spindle-shaped product appeared on the surface when the 2205 DSS was immersed in a 3.5 wt. % NaCl solution under a hydrostatic pressure of 10 MPa (corresponding to a seawater depth of 1000 m) for a long period of time. In addition, this product only appeared in the ferrite phase, instead of the austenite phase. This phenomenon can also be observed during electrochemically hydrogen charging, causing more product to be generated. Such an interesting phenomenon arouses great attention. Firstly, does this product affect the mechanical properties of 2205 duplex stainless steel (DSS)? Secondly, what is it, and why does it only appear in the ferrite phase? Therefore, the purpose of this paper is to clarify the above-mentioned issues.

Szummer et al. [18,19] reported that some needle-shaped products appeared on the surface of ferritic stainless steel after hydrogen charging. Initially, they thought it was micro-twin, but later proposed that the product could be hydrides. Örnek et al. [20] demonstrated that a hydrogen charging ‘blister’ generates on the ferrite phase of the 2205 DSS surface. However, the common consensus is that the hydrogen blister is usually circular or nearly circular in shape [21,22], but the product morphology observed by us and Örnek was needle- or spindle-shaped. This proves that this hydrogen charging product is highly unlikely to be a hydrogen blister. Unfortunately, a systematic characterization on this product is still absent.

In the present work, the spindle-shaped product was produced in 2205 DSS by electrochemical hydrogen charging; its effect on the mechanical properties was measured by slow strain rate tensile (SSRT) and nanoindentation tests. Afterwards, the product was characterized by a series of experiments. First, the microscopic morphology was observed by atomic force microscopy (AFM) and transmission electron microscopy (TEM), then the composition and crystalline structure were characterized by X-ray diffraction (XRD), energy dispersive spectroscopy (EDS) coupled with scanning electron microscopy (SEM), and secondary ion mass spectrometry (SIMS). Last, the thermal stability of the product was measured by the differential scanning calorimeter (DSC) method. 

## 2. Materials and Methods

### 2.1. Hydrogen Charging Product

The material employed in this study was a commercial 2205 DSS with the following chemical compositions: 0.03 wt. % C, 0.18 wt. % N, 4.83 wt. % Ni, 21.83 wt. % Cr, 0.48 wt. % Si, and balanced Fe. Block samples with a size of 10 mm × 10 mm × 3 mm were used for electrochemical hydrogen charging and surface observation. Samples were wet ground with SiC paper up to 5000 grit, mechanically polished, and then electropolished with nitric acid (50%) at 1.2 V for 30 s. Electrochemical hydrogen pre-charging was performed in a NaOH solution for 48 h at a current density of 2 mA/cm^2^. 

**Figure 1:**
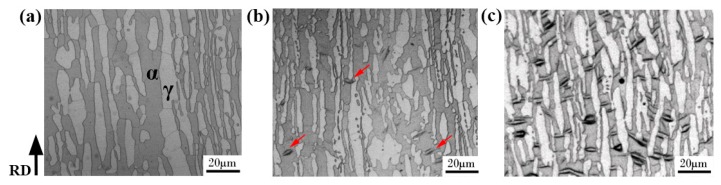
Microstructure of 2205 duplex stainless steel (DSS) for the hydrogen-free sample (**a**), immersion in 3.5 wt. % NaCl solution under 10MPa for 720 h (**b**), and after electrochemical hydrogen charging (**c**).

The microstructure of the hydrogen-free specimen, immersed in 3.5 wt. % NaCl solution under 10 MPa for 720 h and hydrogen pre-charging specimens are shown in Figure 1. Figure 1a displays the morphology of the hydrogen-free sample, and the ferrite and austenite phases are marked. As can be observed from Figure 1b, a spindle-shaped product (indicated by red arrows) appeared in the ferrite phase after immersion in 3.5 wt. % NaCl solution under hydrostatic pressure of 10 MPa for 720 h. interestingly, this spindle-shaped product was reproduced in a larger quantity under electrochemical hydrogen charging conditions (see Figure 1c). It is noteworthy that the product layer was very thin and disappeared after the surface thickness was reduced by about 5 μm by grinding with SiC paper. A detailed microstructural characterization of the spindle-shaped product was subsequently done using AFM (Dimension Icon, Bruker, Billerica, MA, USA) and TEM. The TEM sample was wet-ground to about 40 μm in thickness and then cut into a small wafer with a diameter of 3 mm. Hydrogen charging was followed by a twin-jet electrolytic polishing process. FEI (Hillsboro, OR, USA) G2 F20 TEM with an electron acceleration voltage of 200 kV was used to observe the hydrogen charging product.

### 2.2. Mechanical Properties

Flat tensile specimens with a gauge length of 20 mm, a gauge width of 5 mm, and a thickness of 0.8 mm were machined with SiC paper to 5000 grit, and all the tensile samples were electrolytically polished prior to the SSRT tests (L10-1, LETRY, Xi’an, China). Hydrogen pre-charging was subsequently carried out in a 0.2 mol/L NaOH solution at a charging current density of 2 mA/cm^2^ for 120 h. SSRT tests of the hydrogen pre-charged specimens were conducted under strain rates of 10^−3^ s^−1^, 10^−4^ s^−1^, and 10^−6^ s^−1^, and the hydrogen-free sample was under 10^−6^ s^−1^. The strain was recorded from the cross-head displacement. Finally, SEM was used to observe the sample surface morphology after fracture. 

The micromechanical properties of the hydrogen charging product were also measured. A Hysitron (Eden Prairie, MN, USA) TI 950 Nano-indenter was utilized to test the nano-hardness and reduced modulus of the hydrogen charging product and the 2205 DSS matrix at a peak load of 2500 μN. Both the nano-hardness and reduced modulus tests were repeated at least 10 times.

### 2.3. Characterization of Composition and Crystalline Structure of Hydrogen Charging Product

The Fe, Cr, Mo, and Ni element ratio in austenite, ferrite, and hydrogen charging product area was characterized using EDS (Octane SDD, Apollo XLT SDD, EDAX, USA) coupled with SEM (ZEISS, EVO MA10/LS 10, Oberkochen, Germany). In addition, a SIMS (ToF-SIMS V, ION-TOF, Münster, Germany) test was used for the hydrogen atom distribution analysis. Moreover, a SMARTLAB X-ray diffractometer (XRD, SMARTLAB. 9, Rigaku, Tokyo, Japan) with an angular velocity of 0.2°/s and a tube voltage of 40 kV was used to determine the phase composition of the 2205 DSS.

### 2.4. Thermal Stability Analysis

A cylindrical specimen with a radius of 3 mm and a height of 3 mm was prepared for the DSC (TG-DTA 8121, Rigaku, Tokyo, Japan) tests to measure the phase decomposition temperature. Hydrogen charging was then carried out under the same conditions as the surface observation sample. The test temperature interval was from 150 to 400 °C with heating rate of 10 °C/min. The sample was scanned repeatedly.

### 2.5. Supplementary Experiment

According to the results of DSC and TEM, the product will decompose at 268 °C. Therefore, in order to study the reversibility of the mechanical properties after removing the product, the tensile specimen was heated at 300 °C for 0.5 h to eliminate the product after hydrogen pre-charging, and then SSRT was done under a strain rate of 10^−3^ s^−1^. The method of hydrogen pre-charging is the same as in the previous mechanical properties test used.

## 3. Results

### 3.1. Mechanical Properties

**Figure 2:**
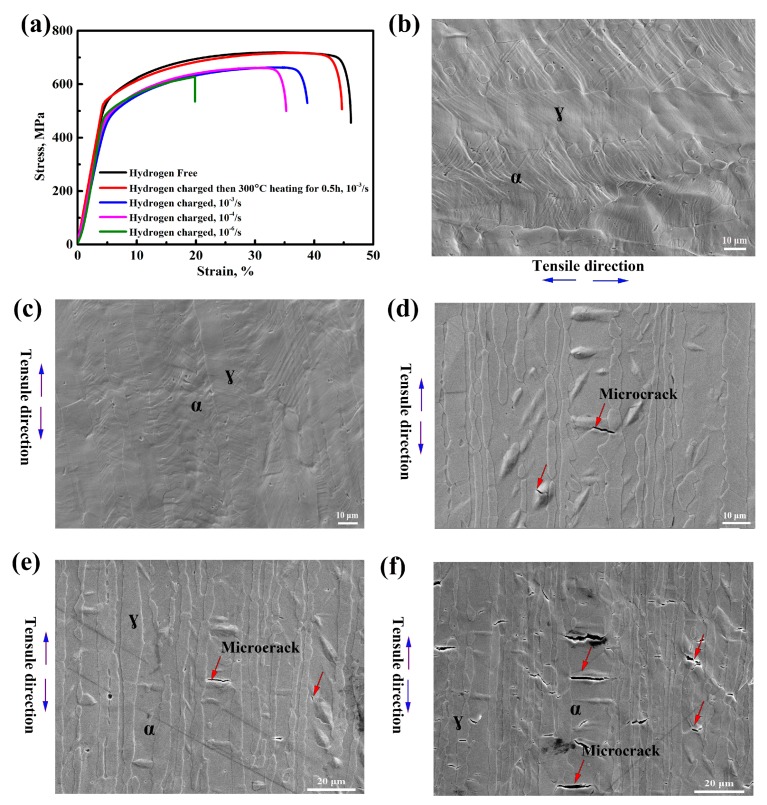
The nominal stress-strain curves of without hydrogen and with hydrogen under different strain rates (**a**); Sample surface for the hydrogen-free sample after fracture (**b**), after heating at 300 °C to remove the product then tensile at 10^−3^ s^−1^ (**c**), and for the hydrogen charging specimens at 10^−3^ s^−1^ (**d**), 10^−4^ s^−1^ (**e**), and 10^−6^ s^−1^ (**f**). The microcracks are indicated by the red arrows.

Figure 2 shows the nominal stress-strain curves and sample surface morphology after fracture of the hydrogen-free and hydrogen pre-charged specimens. As can be observed from Figure 2a, the hydrogen pre-charged specimen showed a remarkable elongation loss compared to the hydrogen-free ones. At high strain rates of 10^−3^ s^−1^ and 10^−4^ s^−1^, the plasticity was significantly reduced after hydrogen charging, as shown by the blue and pink lines in Figure 2a. At a strain rate of 10^−6^ s^−1^, the plastic loss was more serious, as seen by the green line. The elongation loss increased with the decrease in the strain rate. However, when the sample was hydrogen pre-charged and tensile after holding at 300 °C for 0.5 h, the elongation basically recovered to the same level as the hydrogen-free sample, represented by the red line. The sample surface after fracture was then observed. Figure 2b exhibits the sample surface morphology of the hydrogen-free sample, and no micro-cracks appear on the sample surface. Figure 2c displays no micro-cracks and the hydrogen charging product appeared on the sample surface after being heated at 300 °C for 0.5 h. Figure 2d–f show the sample surface morphology of the samples pre-charged and tensile at the strain rates of 10^−3^ s^−1^, 10^−4^ s^−1^ and 10^−6^ s^−1^, respectively. Several micro-cracks (indicated by red arrows in Figure 2d,e) were initiated at the interface of the hydrogen charging product and ferrite matrix at a high strain rate. When the tensile rate was decreased to 10^−6^ s^−1^, a large number of micro-cracks (as seen in Figure 2f) were observed in the ferrite phase. What is more, the micro-cracks initiated not only at the interface between the product and the ferrite matrix, but within the product. 

**Figure 3:**
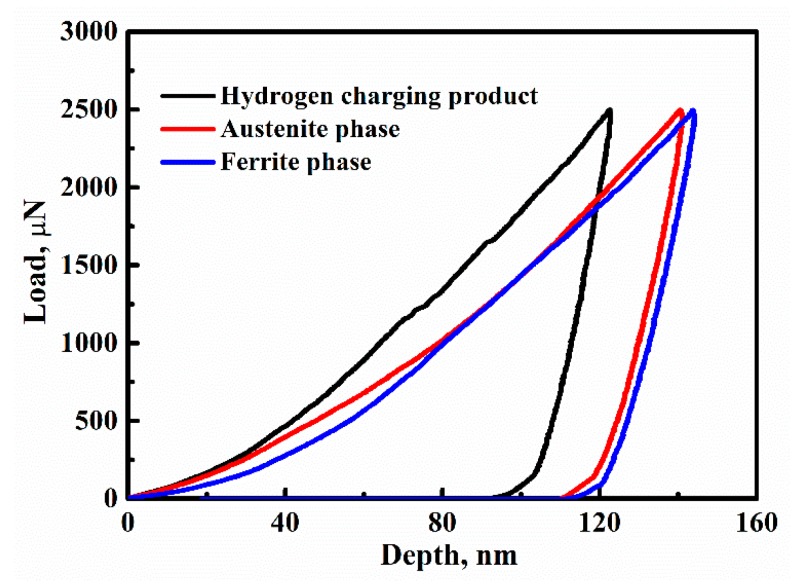
Typical load-depth curves for the three regions of the hydrogen charging product, austenite, and ferrite phases of 2205 DSS.

Figure 3 exhibits the typical load-depth curves of nano-indentation test for the three regions of the hydrogen charging product, austenite, and ferrite phases. The indention depth of the hydrogen charging product area is about 120 nm. After calculation, the indentation size was less than 1 μm^2^. Because the size of the hydrogen charging product is bigger than that of the indentation, the indentation tip could be completely applied to the product. Therefore, the influence of the ferrite matrix on the nano-hardness of hydrogen charging products can be neglected. The values of the average nano-hardness and reduced modulus of the three regions are given in Table 1. The nano-hardness and reduced modulus of the hydrogen charging product were higher than those of the ferrite matrix. The average nano-hardness values of the ferrite and austenite phases were found to be 4.94 and 4.76 GPa, respectively. However, the average nano-hardness of the hydrogen charging product was 6.52 GPa. Moreover, the reduced modulus of the product area (232.4 GPa) was greater than that of the ferrite (190.6 GPa) and austenite (176.7 GPa) phases. Generally, the higher the reduced modules, the more difficult it is to deform. The reason why micro-cracks initiated at the interface between the hydrogen charging product and ferrite is that the deformation inhomogeneity effect caused by the difference of reduced modulus between them. The experimental results of macro and micro mechanical properties show that this product deteriorated the mechanical properties of 2205 DSS and was the origin of the hydrogen-induced cracks. Therefore, it is imperative to identify what the hydrogen charging product is.

### 3.2. Microscopic Morphology of Hydrogen Charging Product

**Figure 4:**
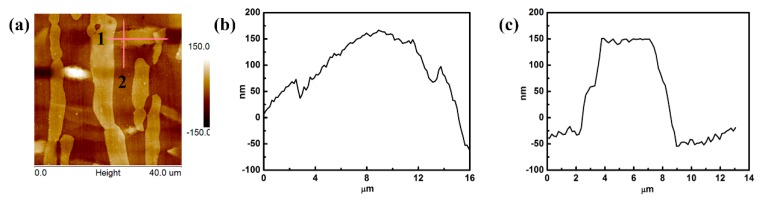
Atomic force microscopy (AFM) morphology of 2205 DSS after hydrogen charging (**a**), and the height contour of lines 1 (**b**), and 2 (**c**).

Figure 4 presents the AFM morphology of 2205 DSS after hydrogen charging. Figure 4a shows the pattern of the scanning area. The dark region represents the ferrite phase, the white areas in the ferrite phase represent the hydrogen charging product, and the other regions correspond to the austenitic phase [23]. The products were spindle-shaped and formed only in the ferrite phase. The length of the products (about 10 to 20 μm) was almost equal to the width of the ferrite phase, while the width of the products was at least a few micrometers. Figure 4b, c shows the height contours of lines 1 and 2 (in Figure 4a), respectively. The products were convex with a circular arc in the horizontal direction (Figure 4b), and flat in the vertical direction (Figure 4c). The maximum height of the product was about 200 nm higher than that of the ferrite matrix.

### 3.3. Composition and Crystalline Structure of Hydrogen Charging Product

Table 2 presents the chemical composition of the hydrogen charging product, ferrite, and austenite phases. The Fe, Cr, and Mo contents in the product were similar to those in the ferrite phase but higher than those in the austenite phase. However, the Ni content in the area with the hydrogen charging product was higher than in the normal ferrite area. 

**Figure 5:**
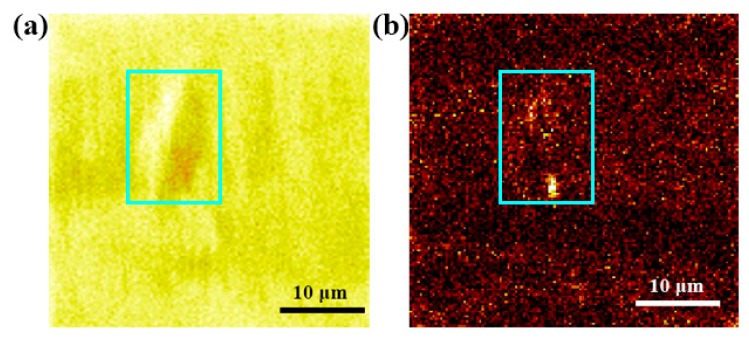
Hydrogen distribution in the hydrogen charging product area. (**a**) Total element content of secondary ion mass spectrometry (SIMS), and (**b**) hydrogen distribution map.

Figure 5 shows hydrogen atom distribution in the hydrogen charging product area. The total element content of the SIMS experiment is given in Figure 5a, while Figure 5b shows the corresponding hydrogen atom distribution in the test area. The light blue rectangles in Figure 5a,b represent the areas with the hydrogen charging product. The brighter the color, the higher the hydrogen concentration. As seen in Figure 5b, the light blue area is brighter than the other areas. That is to say, the hydrogen concentration of the hydrogen charging product is higher than that of the other region.

**Figure 6:**
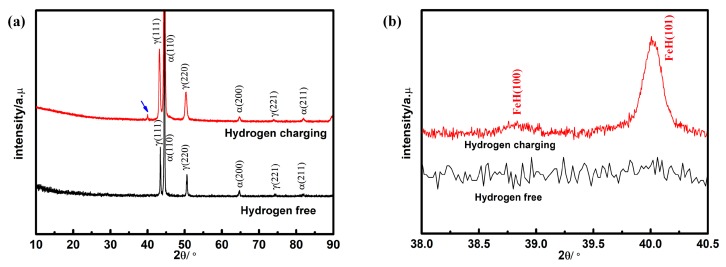
X-ray diffraction (XRD) results of 2205 DSS with and without hydrogen; (**a**) for 2θ ranging from 10° to 90°; (**b**) enlarged region of 38°to 40.5°.

The XRD results of hydrogen-free and hydrogen charging specimens are given in Figure 6. The hydrogen pre-charged specimen showed an additional peak (indicated by the blue arrowhead) between 40 and 41°, as illustrated in Figure 6a. Figure 6b shows an enlarged region near the additional peak. It can be observed that the hydrogen pre-charged specimen showed two additional characteristic peaks at 38.7° and 40.07°, which corresponds to the characteristic peak of iron hydride (FeH) [24], marked in Figure 6b. In connection with the EDS and SIMS analysis, this product is likely to be a hydride of FeH or (Fe, Ni)H.

### 3.4. Decomposition of Hydrogen Charging Product

**Figure 7:**
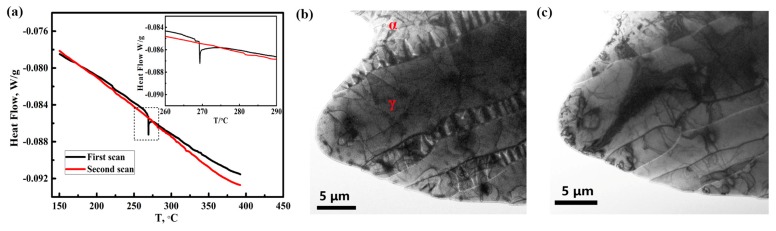
Differential scanning calorimeter (DSC) curves of hydrogen charging specimen, the insert is the local enlarged graph (**a**); in situ transmission electron microscopy (TEM) morphology of the specimen with hydrogen charging product (**b**) which disappeared after the electron beam focusing during TEM observation (**c**).

A DSC analysis was carried out to study the thermal stability of the hydrogen charging product. Figure 7a shows the DSC curves and local enlarged graph for the sample after hydrogen pre-charging. The phase transition peak was observed at 268 °C during the first scan. However, no phase transition peak was observed during the second scan. This indicates that the product was completely decomposed during the first scan test. 

The TEM experiment was originally designed to observe the microstructure of the hydrogen charging product, but the product had decomposed during the electron diffraction focusing process. Figure 7b and c shows the in situ morphology of the specimen with hydrogen before and after irradiation with the TEM-focused electron beam. Prior to the focused electron beam irradiation, as seen in Figure 7b, the hydrogen charging product was distributed uniformly in the ferrite phase, but the product was incomplete, which can be attributed to the hydrogen charging on the thin film sample. However, Figure 7c shows that all the hydrogen charging product disappeared after the electron beam focusing. This is because of the high temperature caused by the focused electron beam. The TEM results demonstrated that the charging product decomposed easily and coincided with the DSC analysis. Therefore, all the experimental results demonstrated that the hydrogen charging product formed in the ferrite phase of 2205 DSS is hydride.

## 4. Discussion

### 4.1. Effect of Hydride on the Mechanical Properties

As shown in Figure 2a, the mechanical properties of 2205 DSS obviously deteriorated after hydrogen pre-charging. The micro-cracks generated during the SSRT test were mainly initiated at the interface of the hydride and ferrite matrix or within the hydride itself, as shown in Figure 2d–f. Previous studies suggest that the formation of hydride could result in brittle cracking [25,26]. On the one hand, the hydrides and matrix are incoherent; their bonding strength with the matrix is low and therefore micro-cracks are initiated under stress. On the other hand, the hydride is a brittle phase itself. Hence, the micro-cracks will also initiate inside the product itself. At high strain rates, the material fractured rapidly, and the effect of hydrogen accumulation is not reflected. In this case, the impact of hydride on mechanical properties is the main factor. At slow strain rates of 10^-6^/s, the effect of hydrogen atoms on elongation loss is more significant, and the elongation is smaller than that at high strain rates, which illustrates that the role of hydrogen atoms is not negligible, in addition to the influence of hydride. The reduction of elongation is caused by the coupled effect of hydride and hydrogen atoms. After removing the hydride and then tensile at 10^−3^ s^−1^, no micro-cracks were observed on the sample surface that are exhibited in Figure 2c, and the elongation basically recovered to the level of the hydrogen-free sample. In the presence of hydride, the mechanical properties of 2205DSS decreased, and the mechanical properties recovered when the hydride disappeared. As a result, it can be stated that this hydride directly deteriorated the mechanical properties of 2205 DSS at 10^−3^ s^−1^.

### 4.2. The Formation of Hydride in Ferrite Phase

The SIMS, XRD, and DSC results reveal that the hydrogen charging product formed in the ferrite phase was hydride. Hydride can be found easily in metals such as titanium, zirconium, and vanadium after hydrogen charging at room temperature, as hydrogen dissolves in these metals in an exothermic reaction; thus, the hydrogen solubility of these metals increases with a decrease in the temperature [25,27]. However, for some metals, such as iron and copper, the absorption of hydrogen causes endothermic reactions, which means there is no critical hydrogen concentration for the formation of hydride at room temperature. Therefore, it is difficult for the hydride to be generated in ferritic steel. Researchers have also found that hydrogen damage similar to a hydrogen bubble occurs in ferritic steel under high temperature and pressure. However, this is different from our study, which was carried out at room temperature [28]. Reed and Holzworth [29,30] claim that hydride appears in the austenite phase of steel materials, such as Fe-Ni systems, but this hydride transforms into the martensite phase at room temperature. Unfortunately, there are no experimental reports on the formation of hydrides in the ferrite phase of steel material under normal temperature and pressure. In particular, computational simulations have revealed that FeH formed at the stress concentration locations [31,32]. In addition, it has been reported that FeH with a double hexagonal close-packed structure is formed in pure iron under extreme conditions such as at hydrogen pressure higher than 3.5 GPa [33]. According to the Sievert equation, hydrogen pressure also exists on the surface of a material during hydrogen charging [25]. The relationship between the hydrogen content and the hydrogen pressure can be expressed as follows:

(1) $$C_{H}=A\sqrt{P}e^{-\Delta H/RT}$$

(2) $$A=e^{\Delta S/R}$$

where *C_H_* is the surface hydrogen content, P is the surface hydrogen pressure during hydrogen charging, *R* is the ideal gas constant, and ΔH and ΔS correspond to the enthalpy change and entropy change of the hydrogen dissolved in the metal, respectively. For solid materials, ΔS≈0 and A≈1. According to Dagbert’s study [34], for Fe-Ni-C (face-centered cubic) systems, the Sievert equation can be simplified as follows:

(3) $$C_{H}=20\sqrt{P}e^{-825/T}$$

The hydrogen diffusion coefficient of the 2205 DSS is in the range of 10^−12^ to 10^−10^ cm^2^/s [35]. It is calculated that the hydrogen diffusion distance was tens of micrometers after hydrogen charging for 48 h. Due to the high hydrogen solubility and slow diffusion coefficient of the austenite phase, all hydrogen atoms would accumulate in the tens of microns zone beneath the sample surface. When the *C_H_* reaches 100 ppm, the surface pressure p can be as high as 1 GPa. Kiuchi [36] calculated the surface hydrogen pressure of several types of steel under various conditions and found that a surface pressure of 5 GPa was obtained when the steel was charged in a H_2_SO_4_ solution with As_2_O_3_ as the promoter. Hence, under electrochemical hydrogen charging conditions, the hydrogen pressure level could reach the criteria for FeH formation. Moreover, the solubility of hydrogen in the ferrite phase is lower than that in the austenite phase. It is also found that the ferrite phase is encapsulated by the austenite phase (as seen in Figure 1), so that the small hydrogen diffusion coefficient of the austenite phase hinders the diffusion of hydrogen from ferrite into the austenite phase and then promotes the supersaturation of hydrogen in the ferrite phase. Hydride is produced when the hydrogen content of the ferrite phase exceeds the ultimate solubility limit for hydride formation. As seen in Figure 5 and Figure 6, and Table 2, H and Ni accumulated in the hydrogen charging product region. The additional characteristic XRD peak observed in the hydrogen-charged specimen corresponds to the FeH phase. Thus, it can be deduced that this hydride was FeH or (Fe, Ni)H. Moreover, the height of the hydrogen-charging product was about 200 nm higher than that of the ferrite phase, as shown in Figure 3. This can be attributed to the incoherence of the hydride and the metal matrix [37]. Hydride is a phase that is easy to decompose [26]. In this study, the hydride decomposition temperature is 268 °C. Due to the instability of FeH, this hydride decomposed under electron beam focusing, as presented in Figure 7c. An earlier report recognized the hydrogen charging product in the ferrite phase as micro-twins [18]. However, the formation of twins is related to the stacking fault energy (SFE), and twins are hardly generated in the high SFE phase. The SFE of the austenite phase of DSS is in the range of 20 to 40 mJ/m^2^, while that of the ferrite phase is greater than 200 mJ/m^2^ [38]. Therefore, it is difficult to form twins in the ferrite phase. Silverstein et al. [39] also observed the same kind of hydrogen charging product. However, neither of them devoted attention to characterizing this product. In this study, we not only obtained evidence to certify that the product produced in the ferrite phase of 2205 DSS is hydride, but also demonstrate such hydride seriously deteriorated that the mechanical properties. Thus, our future work will focus on this hydride induced hydrogen embrittlement of 2205 DSS.

## 5. Conclusions

A spindle-shaped hydrogen charging product was found in the ferrite phase of 2205 DSS. The following conclusions are drawn from this work:

(1) Once the hydrogen charging product is formed, the elongation of 2205 DSS will decrease severely. The microcracks were initiated at the interface of the hydrogen charging product and ferrite matrix and the hydrogen charging product itself.
(2) The hydrogen charging product can be observed by TEM but will be decomposed due to the high temperature caused by electron beam focusing. A DSC test shows that the decomposition temperature of the hydrogen charging product is 268 °C.
(3) The hydrogen charging product only appears in the ferrite phase, which is related to the supersaturation of hydrogen in the ferrite phase. The hydrogen charging product is a hydride of FeH or (Fe, Ni)H.

## Figures and Tables

**Table 1:** Average nano-hardness and reduced modulus of the different phases of 2205 DSS.

Phase	Nano-Hardness, GPa	Reduced Modulus, GPa
Austenite	4.76 ± 0.21	176.7 ± 14.1
Ferrite	4.94 ± 0.32	190.6 ± 11.4
Hydrogen charging product	6.52 ± 0.54	232.4 ± 19.2

**Table 2:** Chemical composition of the hydrogen charging product, ferrite, and austenitic phases areas.

Area	Fe, wt%	Cr, wt%	Ni, wt%	Mo, wt%
Austenite	64.04	23.10	6.27	3.26
Ferrite	63.06	25.67	3.81	4.68
Hydrogen charging product	62.03	23.89	5.62	4.27
